# Mutual Regulation of RNA Silencing and the IFN Response as an Antiviral Defense System in Mammalian Cells

**DOI:** 10.3390/ijms21041348

**Published:** 2020-02-17

**Authors:** Tomoko Takahashi, Kumiko Ui-Tei

**Affiliations:** 1 Department of Biological Sciences, Graduate School of Science, The University of Tokyo, Tokyo 113-0033, Japan; 2 Department of Biochemistry and Molecular Biology, Graduate School of Science and Engineering, Saitama University, Saitama 338-8570, Japan; 3 Department of Computational Biology and Medical Sciences, Graduate School of Frontier Sciences, The University of Tokyo, Chiba 277-8561, Japan

**Keywords:** RNA silencing, microRNA, endogenous RNAs, IFN response, virus, exogenous RNAs, antiviral response, mammalian cells

## Abstract

RNA silencing is a posttranscriptional gene silencing mechanism directed by endogenous small non-coding RNAs called microRNAs (miRNAs). By contrast, the type-I interferon (IFN) response is an innate immune response induced by exogenous RNAs, such as viral RNAs. Endogenous and exogenous RNAs have typical structural features and are recognized accurately by specific RNA-binding proteins in each pathway. In mammalian cells, both RNA silencing and the IFN response are induced by double-stranded RNAs (dsRNAs) in the cytoplasm, but have long been considered two independent pathways. However, recent reports have shed light on crosstalk between the two pathways, which are mutually regulated by protein–protein interactions triggered by viral infection. This review provides brief overviews of RNA silencing and the IFN response and an outline of the molecular mechanism of their crosstalk and its biological implications. Crosstalk between RNA silencing and the IFN response may reveal a novel antiviral defense system that is regulated by miRNAs in mammalian cells.

## 1. Introduction

RNA silencing is a posttranscriptional gene silencing mechanism that is conserved among diverse organisms and is induced by microRNA (miRNA), an approximately 22-nucleotide (nt) long endogenous non-coding double-stranded RNA (dsRNA) [1]. The human genome encodes > 2000 miRNAs [2], and the expression profiles of miRNAs differ among tissues and cell lines [3]. A number of studies have reported that posttranscriptional regulation of gene expression by miRNAs is an important process, and that deletion or mutation, as well as upregulation or downregulation, of miRNA causes serious diseases including cancer, neurodegenerative diseases, and diabetes [4]. By contrast, the type-I interferon (IFN) response is an innate immune response that is conserved among higher vertebrates and induced by exogenous dsRNAs, such as viral RNAs. The IFN response involves upregulated expression of many IFN-stimulated genes (ISGs), repressing viral replication and damage to virus-infected cells [5].

In mammalian cells, endogenous or exogenous RNAs with typical structural characteristics are accurately recognized by specific types of RNA-binding proteins acting in one of the two pathways [6,7,8,9,10,11,12,13,14,15,16,17,18,19,20,21,22,23,24,25]. This specificity may be the reason that RNA silencing and the IFN response have been considered two independent pathways, despite both being induced by dsRNAs in the cytoplasm. However, recent reports, including ours, have provided a novel perspective that RNA silencing and the IFN response are mutually regulated through protein–protein interactions triggered by RNA virus infection [26,27,28,29,30,31,32,33]. In this review, we provide brief overviews of RNA silencing and the IFN response, and outline the molecular mechanism of their crosstalk with consideration of its biological implications. Crosstalk between RNA silencing and the IFN response is a conserved mechanism specific to mammalian cells that might reveal an unresolved molecular mechanism of antiviral defense in mammalian cells. 

## 2. RNA Silencing Directed by Endogenous miRNAs

### 2.1. Biosynthetic Pathway of Endogenous miRNA and Gene Silencing

**Figure 1:**
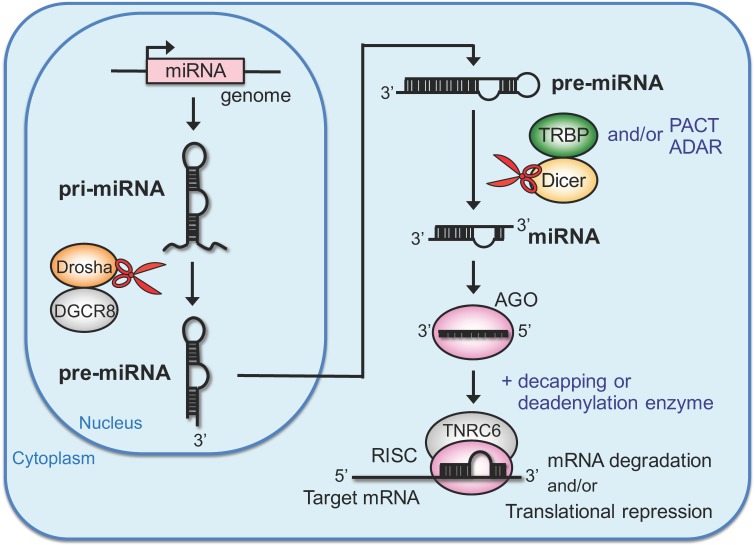
RNA silencing directed by endogenous miRNAs. The primary miRNA (pri-miRNA) is transcribed from the genome and processed into precursor miRNA (pre-miRNA) by the endoribonuclease Drosha in the nucleus. After processing, the pre-miRNA is exported to the cytoplasm and processed into miRNA duplexes by the endoribonuclease Dicer. One strand of the miRNA duplex is bound to Argonaute (AGO) and forms RNA-induced silencing complex (RISC) with trinucleotide repeat-containing gene 6 (TNRC6) and decapping or deadenylation enzymes for mRNA degradation or translational repression of mRNAs.

The human genome encodes 1917 miRNA precursors (pre-miRNAs), from which 2656 mature miRNAs are generated according to miRBase, a searchable database of published miRNA sequences and annotations, release 22 [2]. Higher organisms tend to encode larger numbers of miRNAs compared to simpler organisms, and each tissue or cell type has a typical miRNA expression profile that is related to its specific functions [3]. miRNAs are transcribed by RNA polymerase II from the genome as primary microRNAs (pri-miRNAs) and processed into pre-miRNAs by an endoribonuclease, Drosha, in the nucleus [34] (Figure 1). DiGeorge syndrome critical region gene 8 (DGCR8) interacts with Drosha to form a microprocessor complex in the nucleus that processes pri-miRNA into pre-miRNA [35,36]. DGCR8 recognizes the junction between stem and single-stranded flanking regions of pri-miRNA to assist the processing carried out by Drosha [37]. After the first processing step, pre-miRNAs are exported to the cytoplasm by Exportin-5/Ran-GTP [38,39], and further processed into miRNA duplexes by another endoribonuclease, Dicer [40]. The miRNA duplex is loaded onto the Argonaute (AGO) protein. One strand remains on AGO, while the other strand is eliminated [41]. AGO is a primary component of the effector complex called the RNA-induced silencing complex (RISC) [40,42,43], and various factors including decapping or deadenylation enzymes are recruited into RISC for gene silencing. Trinucleotide repeat-containing gene 6 (TNRC6) is an essential scaffolding protein that recruits other necessary components for mRNA destabilization or translational repression to RISC [44,45,46,47]. The human genome encodes four AGO proteins (AGO1–4) with differing expression levels [40,48]. Essentially, miRNA binds to target mRNAs using sequence complementarity at positions 2–7 or 2–8 from the 5′ end, referred to as the seed region, and induces mRNA degradation or translational repression [10,49]. Among the four AGO proteins, AGO2 has strong slicer activity and AGO3 has weak slicer activity for target mRNA with complementarity in the central region in addition to the seed region [50]. Analysis of the crystal structure of human AGO2 with miRNA revealed that the seed region is bound by AGO2 and the 5′ phosphate of the miRNA is recognized by a pocket of the MID domain [10,11]. Furthermore, a majority of miRNAs contains adenine or uracil at the 5′ end, and this feature is preferable for anchoring in the AGO pocket [51]. The mature miRNA forms base pairs with mRNA on the AGO protein primarily through their 3′ untranslated regions (UTRs) and in some cases through the coding sequence (CDS), but recent reports have shown that miRNAs and AGOs are present in the nucleus and RNA silencing can occur not only in the cytoplasm, but also in the nucleus [52,53,54,55]. These findings may indicate that miRNAs can target introns or non-coding RNAs, which preferably accumulate in the nucleus, in addition to the 3′ UTR or CDS. Thus, a larger number of genes may be targeted by miRNAs both in the nucleus and in the cytoplasm than previously assumed. The miRNA recognizes the target mRNA through complementarity of only 6–7 nucleotides in the seed region [49], indicating that a single miRNA can repress the expression of hundreds of genes [56] and that a single gene may be simultaneously regulated by multiple miRNAs. Thus, miRNAs form a posttranscriptional gene expression system that is systematically regulated.

### 2.2. Modulator Proteins of miRNA Biosynthesis and Gene Silencing in the Cytoplasm

In the RNA silencing pathway in the cytoplasm, multiple essential or important modulator proteins are involved. Trans-activation response (TAR) RNA-binding protein (TRBP) is a dsRNA-binding protein, which interacts with Dicer to enhance processing of pre-miRNA into miRNA duplexes [57,58]. TRBP shows a binding preference for a specific type of miRNA, with stem regions that exhibit tight base pairing, suggesting that TRBP enhances the processing of a subset of miRNAs that bind to TRBP [26]. TRBP may be involved in strand selection of miRNA bound to AGO [59] and the biogenesis of isomiRs, which are miRNA variants generated from shifting of Dicer cleavage sites [60]. Protein activator of protein kinase R (PKR) (PACT) is also a dsRNA-binding protein, and is thought to enhance pre-miRNA processing along with TRBP, but its precise function remains unclear because PACT and TRBP form a heterodimer [61] and the precise composition of the protein complex including Dicer, TRBP, and PACT has not yet been clarified in vivo. An RNA editing enzyme, adenosine deaminase acting on RNA 1 (ADAR1), contains dsRNA-binding domains and an adenosine deaminase domain, and reportedly interacts with Dicer to enhance pre-miRNA processing [62]. The complicated interactions among these dsRNA-binding proteins and endoribonucleases have made it difficult to elucidate the regulatory mechanism of gene silencing.

## 3. IFN Response Directed by Exogenous Viral dsRNAs

### 3.1. Recognition of Exogenous RNAs such as Viral RNAs in the Cytoplasm

**Figure 2:**
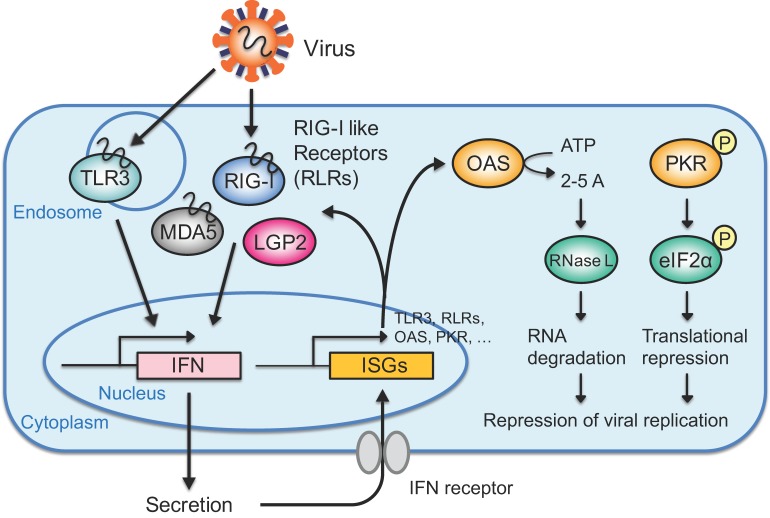
The interferon (IFN) response directed by exogenous RNAs. Exogenous RNAs such as viral RNAs are detected by toll-like receptor 3 (TLR3) in the endosome or retinoic acid-inducible gene I (RIG-I)-like receptors (RLRs) in the cytoplasm. Activated TLR3 or RLRs transfer signals to downstream molecules, inducing IFN production. Among RLRs, laboratory of genetics and physiology 2 (LGP2) does not contain caspase recruitment domain (CARD), which is necessary for signal transfer. The secreted IFN is recognized by the IFN receptor on the cell surface in a paracrine or autocrine manner, inducing the expression of IFN-stimulated genes (ISGs). An ISG, either 2′-5′-oligoadenylate synthetase (OAS) or protein kinase R (PKR), activates RNase L via 2-5A or phosphorylates eIF2α to carry out RNA degradation or translational repression, respectively. Activation of the IFN response represses viral replication and effectively excludes viruses while limiting damage to the cell.

Exogenous dsRNAs, such as viral RNAs, are sensed by host pattern recognition receptors (PRRs), inducing IFN production in mammalian cells. Toll-like receptor 3 (TLR3) [63] and retinoic acid-inducible gene I (RIG-I)-like receptors (RLRs) [64,65] are representative dsRNA sensor proteins for exogenous RNA, known as pathogen-associated molecular patterns (PAMPs) (Figure 2). TLR3 is localized in the endosomal membrane and activates TANK-binding kinase 1 (TBK1) and inducible IκB kinase (IKKi or IKKε) via Toll-interleukin (IL)-1 resistance (TIR) domain-containing adaptor-inducing IFN-β (TRIF) [66,67,68]. RLRs including RIG-I, melanoma differentiation-associated gene 5 (MDA5), and laboratory of genetics and physiology 2 (LGP2) are localized in the cytoplasm [69], and activate TBK1 and IKKi through IFN-β-promoter stimulator (IPS)-1 (also known as MAVS, VISA, or Cardif) on the mitochondrial outer membrane [70,71]. RIG-I and MDA5 contain caspase recruitment domains (CARDs), which are necessary for signal transfer to downstream molecules [69], whereas LGP2 does not contain a CARD and is incapable of transferring signals downstream, leaving its function unclear. Among RLRs, RIG-I and MDA5 are known to recognize different types of exogenous RNAs; RIG-I is activated by 5′-triphosphate- or 5′ diphosphate-containing RNA and small dsRNA [12,13,14,15,16,17,18], whereas MDA5 is activated by long dsRNA [19,20,21,22,23,24,25]. Although the activated RNA molecules in the TLR3 and RLR signaling pathways differ, their activation induces the phosphorylation of interferon regulatory factor (IRF)-3 and IRF-7. Phosphorylated IRF-3 and IRF-7 form a homodimer or heterodimer that is translocated into the nucleus, inducing type-I IFN production [72,73,74]. 

### 3.2. IFN-Mediated Antiviral Response

After production, IFN is secreted from the cell and recognized by IFN-α/β receptor (IFNAR) 1 or IFNAR2 on the cell surface in a paracrine or autocrine manner [75]. Then, IFNAR1 and IFNAR2 activate Janus kinase (JAK)/signal transducer and activator of transcription (STAT) signaling pathways and induce assembly of the tripartite transcriptional factor, IFN-stimulated gene factor-3 (ISGF3), consisting of phosphorylated STAT1 and STAT2 and IRF-9, in the cytoplasm [76,77,78,79,80,81,82]. ISGF3 is translocated into the nucleus, where it induces the expression of ISGs by binding to IFN-stimulated response elements (ISREs) in the promoter regions of ISGs [83]. TLR3 and RLRs are examples of ISGs, and their positive-feedback regulations enhance viral RNA detection [84,85]. The ISGs 2′-5′-oligoadenylate synthetase (OAS) 1, 2, and 3 synthesize 2′-5′-oligoadenylates (2-5A) using adenosine triphosphate (ATP) [86,87], and 2-5A activates RNase L, resulting in RNA degradation [88]. PKR is also a type of ISG, and phosphorylated PKR induces the phosphorylation of eukaryotic initiation factor 2α (eIF2α) and subsequent translational repression [89,90,91]. These RNA degradation and translational repression processes repress viral replication, effectively repressing viruses and damage to virus-infected cells.

## 4. Crosstalk between RNA Silencing and the IFN Response

### 4.1. Similarities and Differences between RNA Silencing and the IFN Response

**Figure 3:**
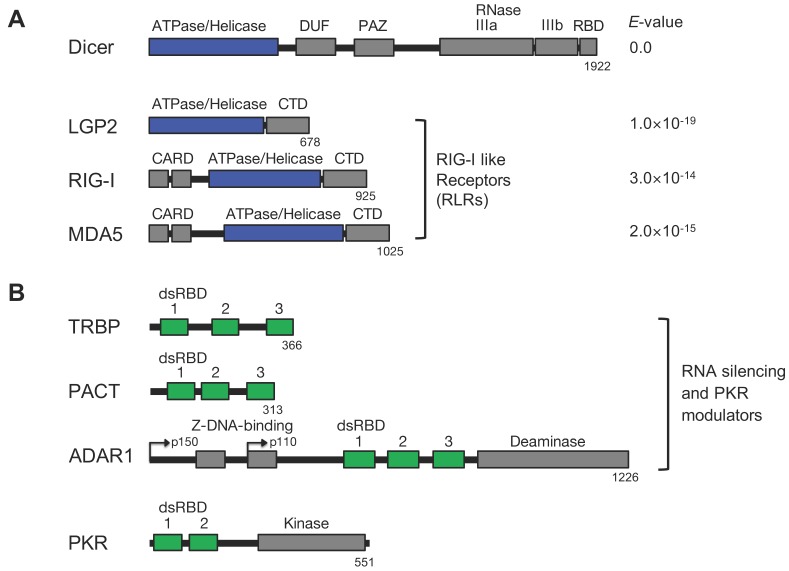
Domain structures of Dicer, RLRs, trans-activation response (TAR) RNA-binding protein (TRBP), protein activator of PKR (PACT), adenosine deaminase acting on RNA 1 (ADAR1), and PKR. (**A**) Domain structures of Dicer and RLRs. *E*-values were calculated using Protein-BLAST compared to the ATPase/helicase domain of Dicer. (**B**) The domain structures of TRBP, PACT, ADAR1, and PKR. TRBP, PACT, and ADAR1 are dual-functional modulators of RNA silencing and PKR activation.

The RNA silencing and IFN response pathways have a few similarities. Dicer is an essential endoribonuclease in RNA silencing, and RLRs are cytoplasmic virus sensors in the IFN response. Both Dicer and RLRs are RNA helicase family proteins containing a DExD/H box that are localized in the cytoplasm. Human Dicer consists of an ATPase/helicase domain, domain of unknown function (DUF), Piwi/Argonaute/Zwille (PAZ), RNase IIIa/b, and an RNA-binding domain (RBD). Both RIG-I and MDA5 consist of two N-terminal CARDs, an ATPase/helicase domain, and a C-terminal domain (CTD), while LGP2 lacks CARDs (Figure 3A). The Protein-BLAST Expect (*E*)-value, which is a parameter describing the number of hits that can be expected by chance in database searches used as a benchmark of sequence similarity, revealed that the amino acid sequences of the ATPase/helicase domains of Dicer and RLRs are similar. In particular, those of Dicer and LGP2 showed strong similarity (Figure 3A). Furthermore, LGP2 interacts with TRBP, a partner protein of Dicer, through the ATPase/helicase domain in addition to its interaction with Dicer, described below. 

Both RNA silencing and the IFN response are induced by dsRNA, although the molecules involved can accurately distinguish between endogenous and exogenous dsRNA. Pre-miRNA is RNA approximately 70-nt in length and typically forms a stem-loop secondary structure with 3′ overhang and 5′-monophosphate, with a stem region that usually includes mismatch(es) and/or bulge(s). Dicer, a pre-miRNA processing enzyme, recognizes the 5′-monophosphate and 3′ overhang of pre-miRNA as a substrate of its PAZ domain [6,7,8]. AGO, a primary component of RISC, recognizes 5′-monophosphate and the 3′ end via its MID and PAZ domains, respectively [9,10,11]. By contrast, RIG-I, a cytoplasmic virus sensor, preferentially recognizes 5′-diphosphate- or 5′-triphosphate-containing RNA and small RNA with blunt ends or 5′ overhangs as exogenous RNAs and initiates signal transfer to downstream molecules for IFN production. RIG-I does not recognize pre-miRNAs or mature miRNAs as exogenous RNAs, as they do not contain 5′-triphosphate or 5′-diphosphate, despite having 5′-monophosphate or 3′ overhang. The other endogenous RNAs ribosomal RNA (rRNA) and transfer RNA (tRNA) are also not recognized by RIG-I, as they do not have 5′-triphosphate or 5′-diphosphate. MDA5, another cytoplasmic virus sensor, recognizes long dsRNAs as exogenous RNAs irrespective of their terminal structures, but such long dsRNAs are rarely produced endogenously in cells. Thus, endogenous and exogenous RNA are distinguished accurately based on their structural characteristics and length by RNA-binding proteins that function in each pathway. 

Furthermore, several mechanisms prevent indiscriminate recognition by the molecules in each pathway. ADAR1 has been reported to edit adenosine to inosine in > 200-nt long dsRNA formed within the 3′ UTR of endogenous mRNAs, destabilizing the dsRNA and preventing indiscriminate recognition by MDA5 as exogenous RNA [92]. Methylation of the penultimate nucleotide (N1), which is commonly found in the vertebrate mRNA cap1 structure (m7GpppNmN-RNA), represses misrecognition by RIG-I and knockdown of cap1 methyltransferase, MTr1, induces the IFN response triggered by RIG-I [93]. These mechanisms prevent indiscriminate activation of the IFN response in mammalian cells in the absence of exogenous stimuli.

### 4.2. Regulation of RNA Silencing by IFN-Stimulated Genes

As noted above, both Dicer and RLRs are DExD/H box-containing RNA helicase family proteins, and the ATPase/helicase domains of Dicer and LGP2 are similar. Dicer interacts with TRBP through its ATPase/helicase domain [94] and our recent research showed that LGP2 also interacts with TRBP through its ATPase/helicase domain, functioning as a modulator of RNA silencing [26]. TRBP is a dsRNA-binding protein containing three dsRNA-binding domains (dsRBD1–3); TRBP binds to pre-miRNA through dsRBD1 and dsRBD2 and interacts with Dicer through dsRBD3 [95,96,97]. Interestingly, LGP2 binds to TRBP primarily through dsRNA-binding sites on dsRBD2, preventing pre-miRNA from binding to TRBP [26]. The dsRNA-binding affinity of TRBP is much higher than that of Dicer [98], suggesting that substrate recruitment by TRBP affects pre-miRNA processing by Dicer. Furthermore, TRBP has a binding preference for specific pre-miRNAs with tight base pairing in their stems, resulting in specific repression of TRBP-bound pre-miRNAs by LGP2. TRBP binds dsRNA with high affinity in a sequence-independent manner, but does not bind single-stranded RNA (ssRNA), supporting the possibility that TRBP has a binding preference for miRNA with tight base pairing in the stem region [96]. When LGP2 binds TRBP in place of pre-miRNAs, Dicer interacts with TRBP but cannot access pre-miRNAs that would otherwise be bound to TRBP. Then, TRBP-bound pre-miRNA maturation is repressed by LGP2, and consequently a number of target genes regulated by TRBP-bound pre-miRNAs are upregulated. Our most recent study showed that LGP2 enhances apoptosis by upregulating apoptosis-regulatory genes through repression of TRBP-bound pre-miRNAs during viral infection [27]. LGP2 is induced by Sendai virus (SeV) infection and inhibits maturation of a typical TRBP-bound pre-miRNA, miR-106b, as well as its subsequent RNA silencing activity. miR-106b targets initiator and executioner caspases (caspase-2, -8, -3, and 7) directly or indirectly, resulting in upregulation of multiple caspases that induce apoptosis of SeV-infected cells [27].

### 4.3. Regulation of the IFN Response and RNA Silencing by Double-Stranded RNA-Binding Proteins

PKR is an IFN-inducible, serine-threonine kinase that can be phosphorylated by binding to dsRNA through N-terminal dsRBD1 and 2 or autophosphorylated through dimerization [99] (Figure 3B). Phosphorylated PKR induces phosphorylation of eIF2α, which is involved in the initiation phase of translation, resulting in translational repression [89,90,91]. TRBP and ADAR1 repress the phosphorylation of PKR [100,101,102,103], whereas PACT enhances it through heterodimer formation [104]. TRBP, ADAR1, and PACT all function as modulators of RNA silencing through interactions with Dicer [57,58,62,105]. These proteins function as RNA-silencing modulators through interactions with Dicer and regulate the IFN response through PKR, showing their complicated nature. 

In addition, TRBP and PACT reportedly interact with LGP2 to regulate IFN production during infection with Theiler’s murine encephalomyelitis virus (TMEV) or encephalomyocarditis virus (EMCV), but not during SeV infection [28,29,30]; this finding contrasts with our result that TRBP-LGP2 interaction occurs during SeV infection [27]. This discrepancy during SeV infection of TRBP-LGP2 interactions may result from differences in viral titer. TMEV and EMCV belong to the family Picornaviridae based on recognition by MDA5, and SeV is in the family Paramyxoviridae, which is recognized by RIG-I. Overexpression of TRBP or PACT increased TMEV- or EMCV-triggered, but not SeV-triggered, IFN promoter activity only when LGP2 and MDA5 were also overexpressed, but did not cause an increase with MDA5 alone [28,29]. The interaction of TRBP with LGP2 is RNA-independent [26], while that of PACT and LGP2 is RNA-dependent [29]. Furthermore, PACT stimulates ATPase activity of RIG-I, enhancing IFN production, during SeV or Ebola virus infection [31,32]. PACT is co-localized with MDA5 in the cytoplasm through polyinosinic-polycytidylic acid [poly(I:C)] transfection, which mimics RNA viral infection and facilitates MDA5 and RIG-I by enhancing oligomerization of MDA5 [33]. Summarizing these findings, the effects of interactions among TRBP, PACT, PKR, and RLRs are highly complex. Further studies are needed to reveal the molecular mechanisms of IFN production regulation by these dsRNA-binding proteins.

## 5. Biological Implications of Crosstalk between RNA Silencing and the IFN Response

Both RNA silencing and the IFN response are induced by dsRNAs in the cytoplasm, but they have been considered two independent pathways in mammalian cells. RNA silencing is a posttranscriptional gene silencing mechanism conserved in diverse organisms. By contrast, the IFN response is an antiviral response of the innate immune system that is conserved among higher vertebrates. The mutual regulation of RNA silencing, directed by endogenous miRNAs, and the IFN response, directed by exogenous RNAs such as viral RNAs, might be a conserved mechanism specific to mammalian cells. Moreover, several proteins with functions in RNA silencing or the IFN response interact with multiple proteins: (i) TRBP interacts with Dicer, PACT, PKR, and LGP2, (ii) LGP2 interacts with RIG-I, MDA5, and TRBP, and (iii) PKR interacts with TRBP, PACT, and ADAR1. RNAs appear to be clearly distinguished as endogenous or exogenous for incorporation into each pathway, irrespective of the complicated interactions among proteins. Endogenous RNAs such as miRNA, mRNA, rRNA, and tRNA do not usually activate the IFN response in human cells, and their misrecognition by virus sensor proteins is prevented by the proteins ADAR and MTr1.

Crosstalk between RNA silencing and the IFN response through protein–protein interactions may have several advantages for mammalian cells: (i) introduction of exogenous viral dsRNA into the cell triggers the crosstalk response through viral sensor proteins, (ii) expression levels of the responsible molecules differ among tissues or cell types, allowing tissue- or cell-specific functions, (iii) such a complicated response may support flexible responses depending on the species of virus, and (iv) the balance of cell survival and death among virus-infected cells can be regulated based on the infecting virus or the expression levels of each molecule. The proteins described above form heterodimers or homodimers in vitro, but it remains unclear whether they form a complex in any combination in vivo. RLRs, PKR, and ADAR1 p150 are IFN-inducible genes, indicating that their expression levels are upregulated during viral infection. By contrast, Dicer, AGO, TRBP, PACT, and ADAR1 p110 are not induced by IFN, but their expression levels are known to differ among types of tissues or cells [106]. Differences in the expression levels of these proteins may strongly impact their interaction patterns and functions. Furthermore, LGP2 induced by IFN upregulates apoptosis regulatory genes during viral infection through repression of TRBP-bound pre-miRNAs. The interaction of TRBP and LGP2 may function as hub proteins in both RNA silencing and RLR signaling depending on their expression levels. This balance may be an important determinant of survival or death of virus-infected cells according to the viral species or number of replicated viruses present.

In plants and invertebrates, antiviral RNA interference (RNAi) directed by viral siRNA functions as an antiviral defense system by cleaving viral RNAs. However, whether antiviral RNAi occurs in mammalian cells remains controversial. Previous reports have shown that IFN represses antiviral RNAi in mammalian cells [26,107,108], although some have noted that RNAi functions as an antiviral defense system in mammalian cells [109,110]. Whether RNAi directed by exogenous dsRNA functions as an antiviral defense system in mammalian cells remains under discussion; IFN at least represses RNAi directed by long dsRNA or short hairpin RNA (shRNA) in human cells [26,107,108]. Previous reports have suggested that antiviral RNAi and the IFN response are mutually exclusive, although RNA silencing directed by endogenous miRNAs is partially repressed by IFN as an antiviral defense system in human cells. The IFN response is a defense system that evolved in higher vertebrates; the reason for its repression of antiviral RNAi in mammalian cells is unclear, as no disadvantage to both the IFN response and antiviral RNAi occurring simultaneously during viral infection is apparent. This question remains unsolved, but antiviral RNAi may effectively combat viruses in virus-infected cells by cleaving viral RNAs. By contrast, IFN can repress viral replication in virus-infected cells and transfer the signal of viral infection from virus-infected cells to non-infected cells as an antiviral cytokine in a paracrine manner. The IFN response may be more effective than antiviral RNAi for multicellular organisms such as human beings. Furthermore, the IFN response is involved in the activation of adaptive immunity through antigen presentation and T cell stimulation by dendritic cells [111]. Prompt clearance of the virus through antiviral RNAi prior to activation of the IFN response in virus-infected cells may inhibit both signal transfer to non-virus-infected cells and the activation of adaptive immunity. Thus, attenuated antiviral RNAi and the repression of RNA silencing directed by a subset of endogenous miRNAs may be advantageous to the antiviral defense system of mammalian cells.

## 6. Conclusions

Recent works from our group and others worldwide have revealed that RNA silencing directed by endogenous miRNAs and the IFN response directed by exogenous dsRNAs such as viral RNAs are a mutually regulated antiviral defense system in mammalian cells. Although the protein–protein interactions involved in RNA silencing and the IFN response are complicated, the elucidation of such interactions may shed light on the newly discovered crosstalk as a common mechanism conserved among mammalian cells. Further studies are needed to reveal the antiviral effects of this crosstalk during viral infection of mammalian cells.
